# Patterns of neural activity in prelimbic cortex neurons correlate with attentional behavior in the rodent continuous performance test

**DOI:** 10.1101/2024.07.26.605300

**Published:** 2024-07-26

**Authors:** Jorge Miranda-Barrientos, Suhaas Adiraju, Jason J. Rehg, Henry L. Hallock, Ye Li, Gregory V. Carr, Keri Martinowich

**Affiliations:** 1 Lieber Institute for Brain Development, Johns Hopkins Medical Campus, Baltimore, MD, 21205, USA; 2 Department of Neuroscience, Johns Hopkins School of Medicine, Baltimore, MD, 21205, USA; 3 Department of Pharmacology and Molecular Sciences, Johns Hopkins University School of Medicine, Baltimore, MD, 21205, USA; 4 Neuroscience Program, Lafayette College, Easton, PA, 18042, USA; 5 Department of Psychiatry and Behavioral Sciences, Johns Hopkins School of Medicine, Baltimore, MD, 21205, USA; 6 The Kavli Neuroscience Discovery Institute, Johns Hopkins University, Baltimore, MD, 21205, USA

## Abstract

Sustained attention, the ability to focus on a stimulus or task over extended periods, is crucial for higher level cognition, and is impaired in individuals diagnosed with neuropsychiatric and neurodevelopmental disorders, including attention-deficit/hyperactivity disorder, schizophrenia, and depression. Translational tasks like the rodent continuous performance test (rCPT) can be used to study the cellular mechanisms underlying sustained attention. Accumulating evidence points to a role for the prelimbic cortex (PrL) in sustained attention, as electrophysiological single unit and local field (LFPs) recordings reflect changes in neural activity in the PrL in mice performing sustained attention tasks. While the evidence correlating PrL electrical activity with sustained attention is compelling, limitations inherent to electrophysiological recording techniques, including low sampling in single unit recordings and source ambivalence for LFPs, impede the ability to fully resolve the cellular mechanisms in the PrL that contribute to sustained attention. *In vivo* endoscopic calcium imaging using genetically encoded calcium sensors in behaving animals can address these questions by simultaneously recording up to hundreds of neurons at single cell resolution. Here, we used *in vivo* endoscopic calcium imaging to record patterns of neuronal activity in PrL neurons using the genetically encoded calcium sensor GCaMP6f in mice performing the rCPT at three timepoints requiring differing levels of cognitive demand and task proficiency. A higher proportion of PrL neurons were recruited during correct responses in sessions requiring high cognitive demand and task proficiency, and mice intercalated non-responsive-disengaged periods with responsive-engaged periods that resemble attention lapses. During disengaged periods, the correlation of calcium activity between PrL neurons was higher compared to engaged periods, suggesting a neuronal network state change during attention lapses in the PrL. Overall, these findings illustrate that cognitive demand, task proficiency, and task engagement differentially recruit activity in a subset of PrL neurons during sustained attention.

## Introduction

Sustained attention, the ability to focus on relevant stimuli or tasks over extended periods, represents a fundamental component of cognitive processes [1]. Deficits in attention are noted in individuals diagnosed with a number of neuropsychiatric and neurodevelopmental disorders, including attention-deficit/hyperactivity disorder (ADHD) [2,3], schizophrenia [4,5,6], and depression [7,8]. These deficits are negatively correlated with functional outcomes and quality of life in patients diagnosed with these disorders. Identifying the cellular and circuit mechanisms mediating sustained attention is a crucial step toward identifying novel pharmacological targets for deficits in attention.

The most commonly used measure to quantify attention in neuropsychological batteries is the continuous performance test (CPT) [9]. The CPT has been used to assess attention deficits in patients with brain damage [10], and in individuals with neuropsychiatric disorders such as schizophrenia [11] and ADHD [12]. A rodent CPT (rCPT) was developed as a touchscreen-based translational version of the human CPT, where mice need to discriminate between a target stimulus (S+) and a non-rewarded stimuli (S−) in a series of successive trials [13,14]. The rCPT has predictive validity [13,14] as pharmacological agents that improve attention (e.g. amphetamine, methylphenidate, and atomoxetine) produce similar effects across species [6,15,16], and homologous brain regions are recruited during performance across species [6,15,16,17].

The human dorsal anterior cingulate cortex (dACC) is strongly linked to sustained attention. Brain imaging studies demonstrated dACC activation in subjects performing attentionally demanding tasks [18,19,20,21,22], including high-load tasks that incorporate presentation of distractors [23]. Moreover, dACC activity is decreased in individuals diagnosed with disorders characterized by sustained attention deficits such as ADHD [3,24], schizophrenia [25,26], and obsessive-compulsive disorder [22]. In rodents, the prelimbic cortex (PrL) shows both functional and anatomical similarity to the human dACC [27,28]. Neuronal activity within the PrL is correlated with attention [17,29,30,31], while inhibiting neuronal activity in the PrL [32] or inputs to the PrL [33], disrupts sustained attention. Moreover, we reported changes in the power and coherence of PrL oscillations, as well as their directionality with locus coeruleus (LC) oscillations during sustained attention in the rCPT [34]. However, how neuronal dynamics at the level of individual PrL neurons underlie attentional behavior remain to be elucidated.

In this study, we used the genetically encoded calcium sensor (GCaMP6f) to conduct *in vivo* endoscopic calcium imaging to investigate neural dynamics in individual PrL neurons in mice performing the rCPT. We trained mice in the rCPT, and recorded calcium activity of PrL neurons at three time points (Stage 2, Stage 3-early, and Stage 3-late), which require differing levels of cognitive demand, and reflect different levels of task proficiency. We identified heterogeneous responses of PrL neurons during behavioral responses - specifically, a higher proportion of PrL neurons were modulated during correct responses, particularly in sessions requiring high cognitive demand during which mice performed at high levels of proficiency (Stage 3-late). Moreover, during rCPT sessions, mice intercalated non-responsive-disengaged periods with responsive-engaged periods that resemble attentional lapses. Analysis of calcium activity in PrL neurons during those periods revealed changes in the correlation of the calcium activity between PrL neurons during engaged and disengaged periods. These results support the notion that attention lapses are associated with unique neuronal states.

## Materials and Methods

### Animals

Male C57BL/6J mice (Strain #000664; The Jackson Laboratory, Bar Harbor, ME) were 8–10 weeks old at the start of the experiment. Mice were group housed (4/cage) in disposable polycarbonate caging (Innovive, San Diego, CA) and maintained on a reverse 12/12 light/dark cycle (lights on at 19:00 hours / lights off at 07:00 hours). Following surgeries for virus injection and gradient-index (GRIN) lens implantation, mice were single housed for the remainder of the experiment. Mice received Teklad Irradiated Global 16% Protein Rodent Diet (#2916; Envigo, Indianapolis, IN) in the home cage ad libitum until the start of the food restriction protocol, and water was available in the home cage ad libitum throughout all experiments. Behavioral testing was conducted Monday-Friday during the dark phase (07:00–19:00 hours). All experiments and procedures were approved by the Johns Hopkins Animal Care and Use Committee and in accordance with the Guide for the Care and Use of Laboratory Animals.

### Surgical procedures

Mice were anesthetized with isoflurane (induction: 2–4% in oxygen, maintenance: 1–2%) and secured to a stereotaxic frame. The top of the skull was exposed by an incision along the midline of the scalp, Bregma and Lambda were identified, and the head was leveled to ensure the skull was flat. A small hole was drilled with a 0.9 mm burr (Fine Science Tools, Foster City, CA) above the PrL, and 400 nl of a viral vector encoding the fluorescent calcium sensor (AAV1.Syn.Flex.GCaMP6f.WPRE.SV40; titer ≥ 1×10¹³ vg/mL) was injected into the PrL (AP: +1.8; ML: ±0.3, DV: −1.4). Injections were made using a Micro4 controller and UltraMicroPump along with a 10 μl Nanofil syringes equipped with 33-gauge needles (WPI Inc., Sarasota, FL). The syringe was left in place for 10 minutes after injection to minimize diffusion. Following viral injections, a gradient-index (GRIN) lens was implanted directly above the PrL. First, a slightly larger hole was drilled with a 1.8 mm burr (Fine Science Tools, Foster City, CA) at the viral injection site. Blood was cleaned using a sterile saline solution and swabbed until bleeding stopped, and the skull hole was clear. Then, a 4.00 mm X 1.00 mm GRIN lens integrated with a baseplate (Inscopix Inc., San Diego, CA) was slowly lowered using the stereotaxic frame at a rate of 0.2 mm / min until it reached 300 μm above the PrL (GRIN lens coordinates: AP: 1.7, MV: ±0.3, DV: −1.5). The GRIN lens was then secured to the skull using three small skull screws Fine Science Tools, Foster City, CA) positioned along the skull for extra support, and black dental acrylic (Ortho-jet, Lang Dental manufacturing Co., Wheeling, IL) to obscure outside light. Following surgery, the incision site not covered by the dental acrylic was closed using surgical staples (Fine Science Tools, Foster City, CA), and animals recovered on a heating pad for 30–60 mins before being returned to the colony room. Animals were closely monitored for health and recovery progress and received Meloxicam injections (20 mg/kg) to relieve pain for three additional days.

### Food restriction protocol

Before initiating behavioral training, mice were subject to a food restriction protocol to increase motivation to perform the task. Briefly, mice were handled and weighed for at least two consecutive days before starting the food restriction protocol. Then, mice were food restricted to 2.5 g of chow per mouse per day and weighed daily to monitor maintenance to 85–90% of their predicted free-feeding weight based on average growth curve data for the strain (The Jackson Laboratory, Bar Harbor, ME). To familiarize the mice to Nesquik^®^ strawberry milk (Nestlé, Vevey, Switzerland), which was used as reward during rCPT training, a 4×4 inch weighing plate (VWR, Radnor, PA, USA) containing ~2 ml of strawberry milk was introduced to the home cage for three consecutive days. The weighing plate was left in the cage until all mice had sampled the strawberry milk.

### Behavioral training

#### Habituation:

Mice were given two consecutive habituation sessions (20 min length) in Bussey-Saksida mouse touchscreen chambers (Lafayette Instruments, Lafayette, IN) to familiarize them to the chambers. In habituation sessions, 1 mL of strawberry milk was placed into the reward tray. The screen was responsive to touch, but touches were not rewarded.

#### Rodent Continuous Performance Test (rCPT):

rCPT training protocol was based on a previously described protocol [35]. Briefly, mice were trained in the touchscreen chambers, which were connected to a computer running ABET II software (Campden Instruments, Loughborough, UK) to track behavioral responses during rCPT sessions.

##### Stage 1:

Mice received 45 min training sessions (Monday-Friday), during which they learned to respond to a visual stimulus (white square) presented at the center of the touch screen. The stimulus was displayed for 10 s (stimulus duration, SD), during which a touch in the center of the screen produced delivery of ~20 ul of strawberry milk in the reward tray located on the opposite side of the chamber. Following SD, a 0.5 s limited hold (LH) period was given in which the screen was blank, but a touch would still yield a reward. Upon interacting with the stimulus, a 1 s tone (3 kHz) was delivered, the reward tray was illuminated signaling reward delivery, and the schedule was paused until a head entry into the reward tray was detected by an IR beam. Then, a 2 s intertrial interval (ITI) would begin before the subsequent trial started. If the mouse did not interact with the stimulus during the SD or LH, an ITI would start, and the next trial would follow. The criterion for a mouse to advance to the next stage was to obtain at least 60 rewards per session in two consecutive sessions.

##### Stage 2:

In Stage 2, a target stimulus (S+) was introduced. The S+ consisted of a square with either horizontal or vertical black and white bars that replaced the white square at the center of the screen. Sessions were 45 min long, and each mouse was assigned either horizontal or vertical oriented S+ for the remaining sessions of the experiment. The S+ assignment was counterbalanced. During Stage 2, the SD was reduced to 2 s, and LH was increased to 2.5 s. Interaction with S+ (hit) during SD + LH resulted in reward delivery. Once a mouse obtained at least 60 hits / session in two consecutive sessions, a recording session (see calcium endoscopic *in vivo* imaging) followed the day after (Stage 2 recording session). A mouse was moved to the next stage if it obtained at least 55 hits in the recording session. If a mouse failed to obtain at least 55 hits in the recording session, the recording session was repeated until the mouse obtained at least 55 hits.

##### Stage 3:

In Stage 3, a non-target stimulus (S−) consisting of a snowflake shape presented at the center of the screen was introduced. On each trial, the probability of S+ / S− was 50% / 50%. The SD and LH were identical to Stage 2, but the ITI length was either 2 or 3 s in length (randomized ITI duration during trials). Similar to Stage 2, screen touches during S+ (hit) yielded a reward but not screen touches during S− (false alarm (FA)). A FA resulted in the beginning of the ITI followed by a correction trial. In correction trials, a S− was presented again. If another FA occurs, a new correction trial starts until the mouse doesn’t interact with the S− (correct-rejection). We used discrimination index (d’) which is a measure of sensitivity bias (refers to the perceptual discriminability between the S+ and S−) to determine attention performance during Stage 3. Mice were trained in Stage 3 until they reached a d’ score of 0.6 or higher for two consecutive days. A recording session occurred during the first Stage 3 session (Stage 3-early) and a second recording session occurred in the following session after two consecutive stage 3 sessions with a d’ of 0.6 or higher (Stage 3-late). In the case that during Stage 3-late a mouse had a d’ under 0.6, the recording session was repeated until they had a session with d’ of 0.6 or higher.

### Behavioral scoring

Behavioral databases containing the timestamps from stimuli presentation, hits, false alarms, latency to response, etc. were retrieved from ABET II (Lafayette Instruments, Lafayette, IN) and Whisker server (Cambridge University Technical Services, UK). Behavioral data was analyzed using Excel to obtain performance scoring parameters. Performance scoring parameters were similar to those described in [13] and [35]. Briefly, to assess attention performance during Stage 3 training, we calculated discrimination index d’ with the following formula:

d’=z(hitrate)-z(FArate)

Whereas:

hitrate(HR)=hits/hits+misses

FArate(FAR)=falsealarms/falsealarms+correctrejections.

### Endoscopic in vivo calcium imaging

Single cell calcium transients were recorded though a GRIN lens coupled to a miniaturized microscope (nVista 3.0, Inscopix Inc. San Diego, CA) at three different behavioral training timepoints: 1) Stage 2; 2) Stage 3-early; and 3) Stage 3-late. Miniscope data was recorded at a 10 Hz frequency rate and the LED power, GAIN, and focus plane were optimized for each mouse. To identify calcium activity from individual neurons from raw miniscope recordings, videos were first preprocessed using Inscopix Data Processing Software (IDPS), Inscopix Inc. San Diego, CA). Videos containing calcium transients were spatially downsampled by a factor of 2, bandpass filtered (0.005 −0.5 pixels), motion corrected, and fluorescent values were normalized using a ΔF / F algorithm. Calcium transients for individual neurons were extracted using principal component analysis (PCA) using the following parameters: average diameter: 15–20 pixels, ICA convergence threshold: 0.00001, ICA temporal weights: 0, ICA max interactions: 100, Block size: 1000, ICA unimix dimension: Spatial. All identified neurons were visually inspected for soma-like morphology (size and shape) and all extracted traces were visually inspected for characteristic dynamics. Neurons with abnormal morphology or with non-consisting calcium transients were rejected for further analysis. Data was exported to the IDEAS platform (Inscopix Inc. San Diego, CA) and a built- in quality control algorithm was used to verify the quality of the extracted traces. For comparing calcium activity surrounding behavioral responses (hits and FA), the Peri-Event-Analysis Workflow v4.3.0 tool in the IDEAS platform was used with the following parameters: Visual window pre: 8 seconds, Visual window post: 8 seconds, statistical window pre-start: 3 seconds, statistical window pre-end: 0 seconds, statistical window post-start: 0 seconds, statistical window post-end: 3 seconds, number of random shuffles: 1000, seed: 0, and significance threshold: 0.05. To determine the calcium activity correlations during behavioral states (responsive and non-responsive) we used the compare neuronal correlations across states v2.1.0 tool.

## Results

### Experimental design and acquisition of task proficiency in the rCPT during in vivo calcium imaging sessions

**Figure 1.**
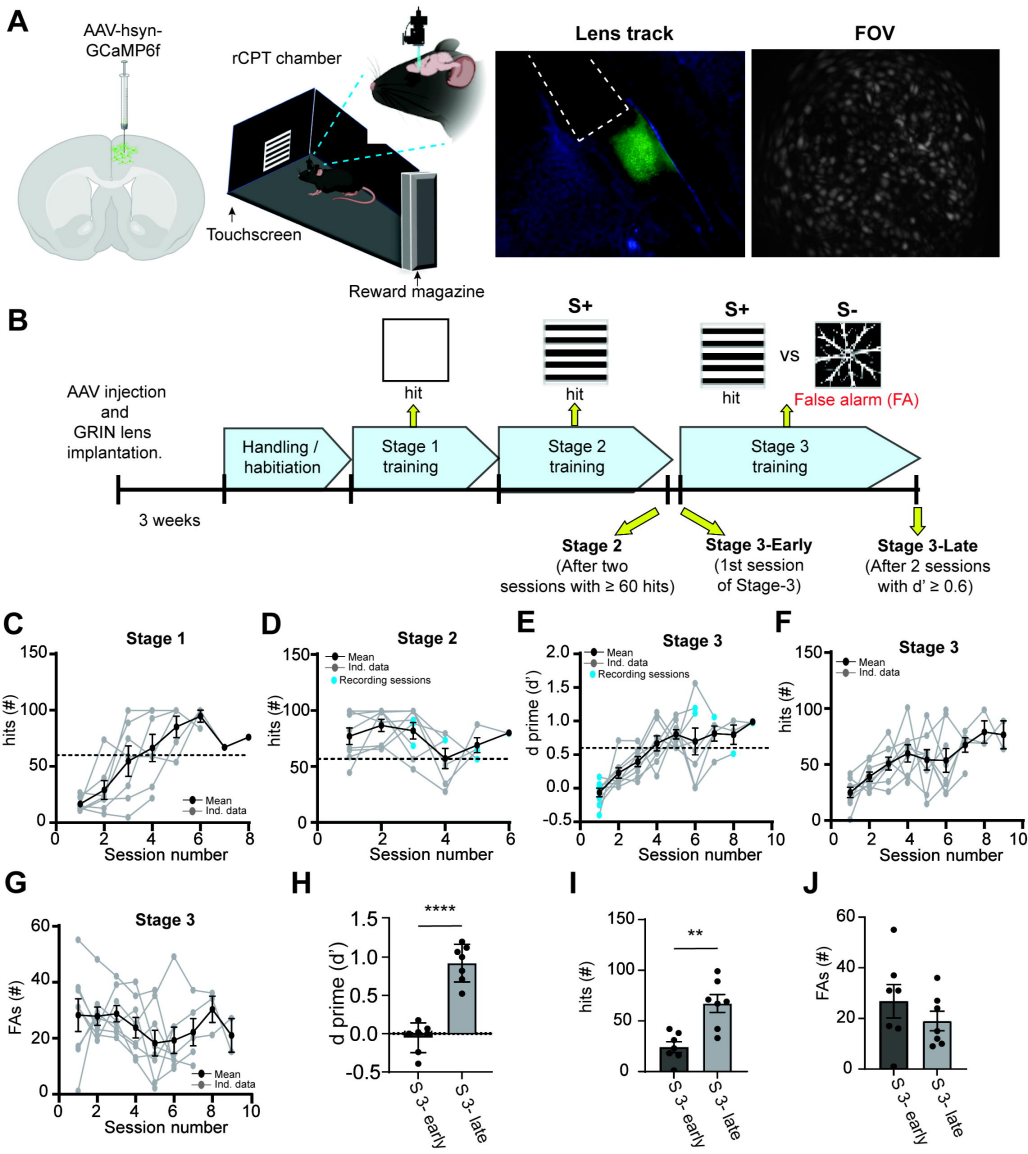
Behavioral performance during rCPT training sessions. A) Schematic of experimental strategy for *in vivo* calcium imaging of PrL neurons during rCPT. B) Timeline for calcium imaging sessions during rCPT behavioral training. C) Number of correct responses (hits) during Stage 1. D) Number of hits during Stage 2. Blue dots represent calcium imaging sessions during Stage 2. E) Behavioral performance (represented as d’) during Stage 3 sessions. Blue dots represent calcium imaging sessions during Stage 3-early and Stage 3-late. F) Time course of the number of hits per session during Stage 3. G) Time course of the number of false alarms (FAs, incorrect responses) per session during Stage 3. H) Bar plots comparing performance between Stage 3-early and Stage 3-late, paired t test: t(6) = 9.64 p˂ 0.0001 (left) and behavioral responses (hits paired t test t(6)= 3.99 p= 0.0072 and FAs paired t test t(6)= 1.066 p= 0.32) (right)

Changes in PrL neuronal activity are linked to sustained attention [30,34,36]. However, the cellular mechanisms in the PrL that contribute to sustained attention are not fully understood. To investigate neuronal activity patterns during sustained attention, we expressed a viral vector encoding GCaMP6f in PrL neurons, and then trained mice on the rCPT. We recorded single cell calcium activity of PrL neurons during three rCPT sessions that differ in cognitive demand and task proficiency: 1) Stage 2: mice are proficient in detecting a visual stimulus (low cognitive demand), 2) Stage 3-early: mice are required to discriminate between two stimuli, but performance is close to chance (high cognitive demand/low proficiency), and 3) Stage 3-late: mice are required to discriminate between stimuli, and performance demonstrates the ability to discriminate between the S+ and S− (high cognitive demand/high proficiency) (Fig. 1A–B). Mice quickly learned to respond to the stimulus presented during Stage 1 (Fig. 1C), and displayed a high number of correct responses in the first Stage 2 session (Stage 2 first session hits: 74.14 ± 7.84, Stage 2 last session hits: 72.57 ± 4.2; Fig. 1D). Mice reached criteria for Stage 2 between the second and third Stage 2 session. A decrease in the number of responses was observed in some mice following tethering, but these mice resumed criteria for performance in the following recording session (Supplementary Fig. 1). As expected, in the first Stage 3 session (Stage 3-early), mice exhibit low task performance (low d’ score, and similar number of correct responses (hits), and incorrect responses/False Alarms (FAs); Fig. 1E–H), but quickly improved, reaching criteria between the 6th and 10th session of Stage 3-late (Stage 3-early d’= −0.05 ± 0.07 vs Stage 3-late d’= 0.92 ± 0.09, Fig. 1E and H Stage 3-early hits= 24.14 ± 5.29 vs Stage 3 late hits= 67.14 ± 8.79, Stage 3-early FAs= 26.85 ± 6.5 vs Stage 3 late FAs= 19 ± 3.81, Fig. 1E–H).

### Increased calcium activity in PrL neurons during correct responses

**Figure 2.**
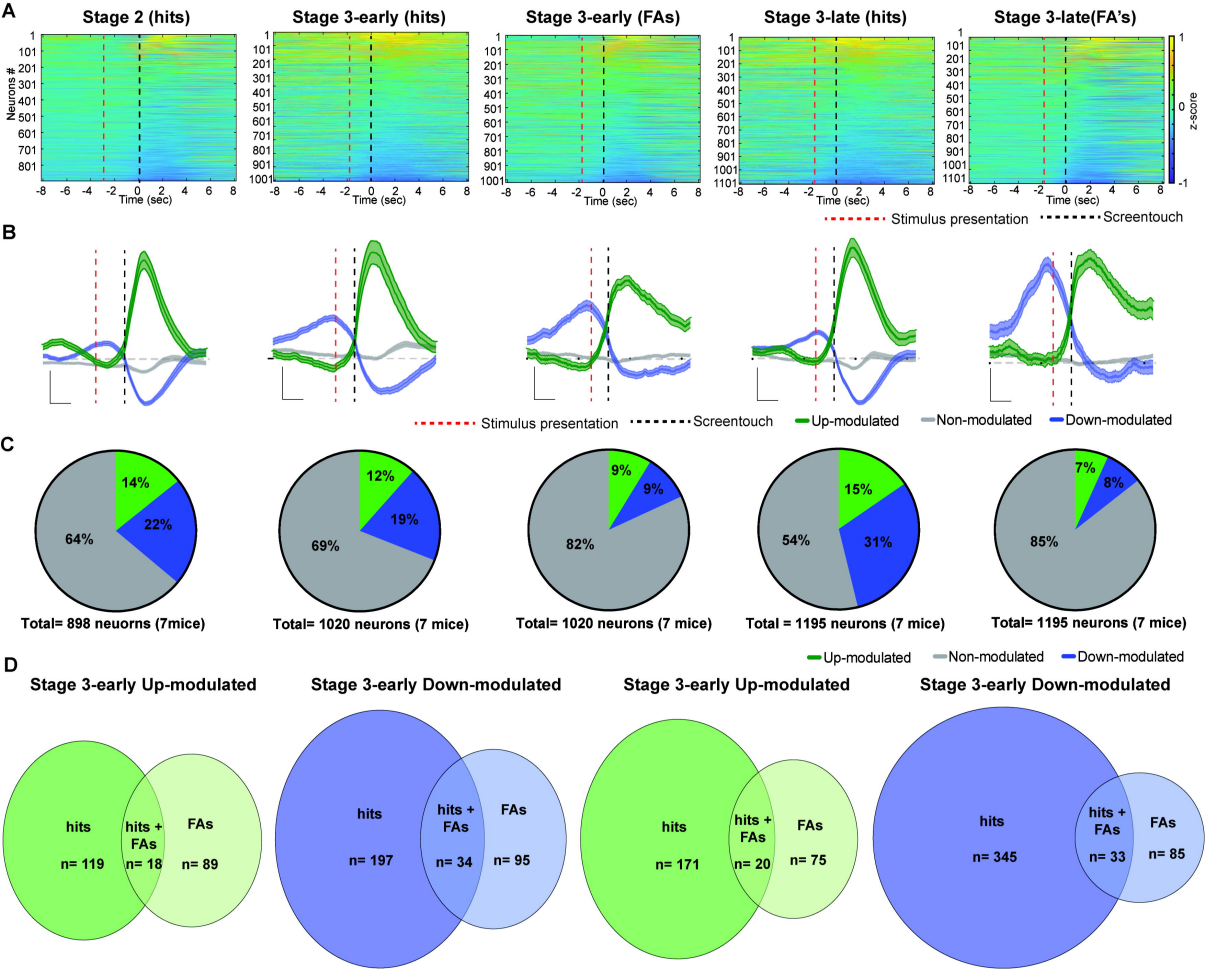
Calcium activity increases in PrL neurons during correct responses. A) Normalized change in calcium activity (z-scored) surrounding hits (correct responses) and false alarms (FAs, incorrect responses) during rCPT recording sessions. B) Calcium activity traces from up-modulated (green), down-modulated (blue) or non-modulated (gray) neurons surrounding the behavioral response. The red dotted line represents the stimulus presentation and the black dotted line represents the behavioral response (screen touch). Scale bar: 2 sec, and z-score= 0.2. C) Pie charts showing the proportion of neurons that were up-modulated (green), down-modulated (blue) or non-modulated (gray) surrounding the behavioral response. D) Venn diagrams showing up-modulated (green) and down-modulated (blue) neurons by hits, FAs, and hits and FAs across Stage 3 recording sessions.

First, we analyzed calcium activity of PrL neurons in time windows surrounding hits or FAs across different rCPT sessions. We found heterogeneous responses across recording sessions - specifically, some neurons were up-modulated while others were down-modulated (Fig. 2 A–C). In Stage 2, 14.2% (128/898) of PrL neurons were up-modulated while 21.8% (196/898) were down-modulated (Fig. 2C). Across both up- and down-modulated neurons, the peak of the averaged calcium transient occurred directly following screen touch (Fig. 2B). However, in a subset of the up-modulated neurons, calcium activity began to increase between the stimulus presentation and screen touch (Fig. 2A), as reflected by a change in the slope of the averaged calcium transient prior to screen touch (Fig. 2B). In stage 3-early, 11.6% (119/1020) of PrL neurons were up-modulated and 19.3% (197/1020) were down-modulated surrounding hits. In contrast, surrounding FAs during stage 3-early, only 8.7% (89/1020) of PrL neurons were up-modulated while 9.3% (95/1020) were down-modulated (Fig. 2C). Similar to Stage 2, the slope of the averaged calcium transient form up-modulated neurons started to change prior to screen touch (Fig. 2B). Moreover, the averaged calcium transient from the down-modulated neurons had a biphasic component where calcium activity increased surrounding the stimulus presentation, followed by a decrease after screen touch (Fig. 2C). During Stage 3-late, 14.3% (171/1195) of PrL neurons were up-modulated while 28.8% (345/1195) were down-modulated surrounding hits (Fig. 2C). In contrast, during FAs, only 6.2% (75/1195) of PrL neurons were up-modulated while 7.1% (85/1195) were down-modulated (Fig. 2C). Comparing the proportion of neurons modulated across responses (hits and FAs) across the three stages, we found that the highest proportion of PrL neurons modulated occurred surrounding hits during Stage 3-late (Fig. 2C). Most of the modulated neurons in Stage 3-early (up-modulated=208/226 neurons, down-modulated=292/326 neurons) and Stage 3-late (up-modulated= 246/266 neurons, down-modulated=430/463 neurons) were specific for behavioral response (hits or FAs) with only a minority of neurons modulating their activity during both hits and FAs (Stage 3-early up-modulated= 18/226, down-modulated=34/326; Stage 3-late up-modulated= 20/266, down-modulated=33/463 neurons) (Fig. 2D).

#### Responsive and non-responsive periods of task engagement are intercalated during the rCPT

**Figure 3.**
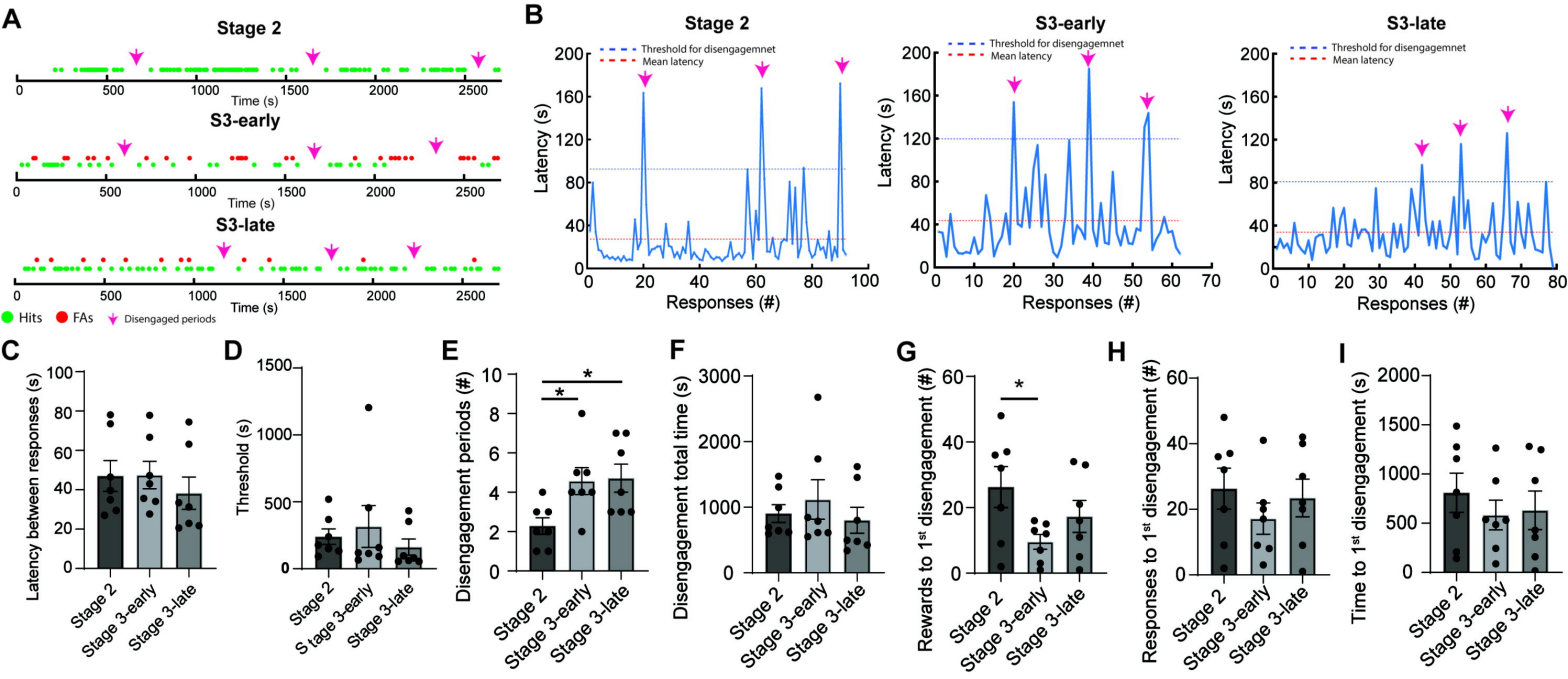
Responsive and non-responsive periods during rCPT behavior. A) Timelines of Stage 2, Stage 3-early, and Stage 3-late sessions showing the pattern of responses of a representative mouse during Stage 2, Stage 3-early, and Stage 3-late. Dots represent the moment where a hit (green) or FA (red) was made. Fiucsia arrows point to disengaged periods during the sessions. B) Plots showing the variability in the latency between two consecutive responses during a Stage 2 (left), Stage 3-early (middle), and Stage 3-late (right) sessions from the same mouse whose patterns of responses were plotted on A. Pink arrows point to peaks of latency between responses that surpassed the threshold for disengagement (blue dotted line; mean of latency +2 SD). C) Average latency between responses during recording sessions during Stage 2, Stage 3-early, and Stage 3-late. One-way ANOVA F= 0.46 *p*= 0.63. D) Threshold for disengagement during Stage 2, Stage 3-early, and Stage 3-late. One-way ANOVA F= 1.33 *p*= 0.29. E) Number of disengaged periods during Stage 2, Stage 3-early, and Stage 3-late. The number of non-responding periods increased during stage 3-early and stage 3-late recording sessions. One-way ANOVA F= 8.67 *p*= 0.006, Tukey’s *post hoc* test Stage 2 vs Stage 3-early *p*= 0.02, Stage 2 vs Stage 3-late *p*= 0.04. F) Total time spent on disengaged periods during Stage 2, Stage 3-early, and Stage 3-late. One-way ANOVA F= 1.24 *p*= 0.32. G) Number of rewards received before the first non-responding period One-way ANOVA F= 3.09 *p*= 0.07, Tukey’s *post hoc* test Stage 2 vs Stage 3-early *p*= 0.04. H) Number of responses (hits and FAs) before the first disengaged period during Stage 2, Stage 3-early, and Stage 3-late. One-way ANOVA F= 0.76 *p*= 0.46. I) Time before the first disengaged period across sessions during Stage 2, Stage 3-early, and Stage 3-late. One-way ANOVA F= 0.55 *p*= 0.58.

A key characteristic of sustained attention is its endurance over time. In humans, sustained attention fluctuates across time resulting in attentional lapses [37]. We hypothesized that attention lapses in the rCPT could be reflected by alterations in task engagement within sessions. To assess changes in task engagement during rCPT behavior, we analyzed response patterns within sessions. Behavioral responses (hits and FAs) followed a heterogeneous distribution across time, with responsive periods intercalated with non-responsive periods of variable length, which were characteristic for individual mice (Fig. 3A and Supplementary Fig. 3). We identified high variability in the latency values within sessions, with peaks that drifted from the mean (Fig. 3B and Supplementary Fig. 4), which coincided with long non-responsive periods. Although the average latency between responses varied between mice, the average response latencies were unchanged across sessions (Stage 2=47.1 ± 7.8 s ; Stage 3-early=47.5 ± 6.9 s; Stage 3-late=38.3 ± 8.2 s) (Fig. 3C), suggesting that the characteristic response patterns for individual mice were stable throughout rCPT training. To further analyze task disengagement during these non-responsive periods, we assigned an arbitrary threshold for the minimum latency between responses (2 standard deviations above the mean; Fig. 3D) to indicate a disengagement period. Using this threshold, the number of disengagement periods increased significantly in Stage 3 sessions compared to Stage 2 (Stage 2= 2.3 ± 0.4; Stage 3-early=4.6 ± 0.7; Stage 3-late=4.7 ± 0.7) ( (Fig. 3E), without affecting the total time spent disengaged (Stage 2= 902.8 ± 136.4 s; Stage 3-early=1117.0 ± 302.0 s; Stage 3-late=801.4 ± 198.1 s) (Fig. 3F). The increase in the number of disengagement periods during Stage 3 sessions could result from increased cognitive demand and subsequent cognitive fatigue in Stage 3, or decreased motivation due to satiety. To determine whether satiety drives disengagement, we analyzed the number of rewards obtained before the first disengagement period across sessions. The number of rewards obtained before the first disengagement was decreased in Stage 3-early compared to Stage 2 (Stage 2=26.3 ± 6.3; Stage 3-early=9.6 ± 2.2; Stage 3-late=17.3 ± 4.9) (Fig. 3 G) without affecting the number of responses (Stage 2= 26.3 ± 6.3; Stage 3-early=17.1 ± 4.8; Stage 3-late=23.4 ± 5.7) (Fig. 3 H) or the time before the first disengagement (Stage 2=804.8 ± 198.6 s ; Stage 3-early=583.1 ± 151.0 s; Stage 3-late=631.6 ± 195.8 s) (Fig. 3I). This result suggests that cognitive demand/fatigue primarily drives disengagement in the rCPT as opposed to effects of satiety.

### PrL calcium activity and state network change during responsive and non-responsive periods.

**Figure 4.**
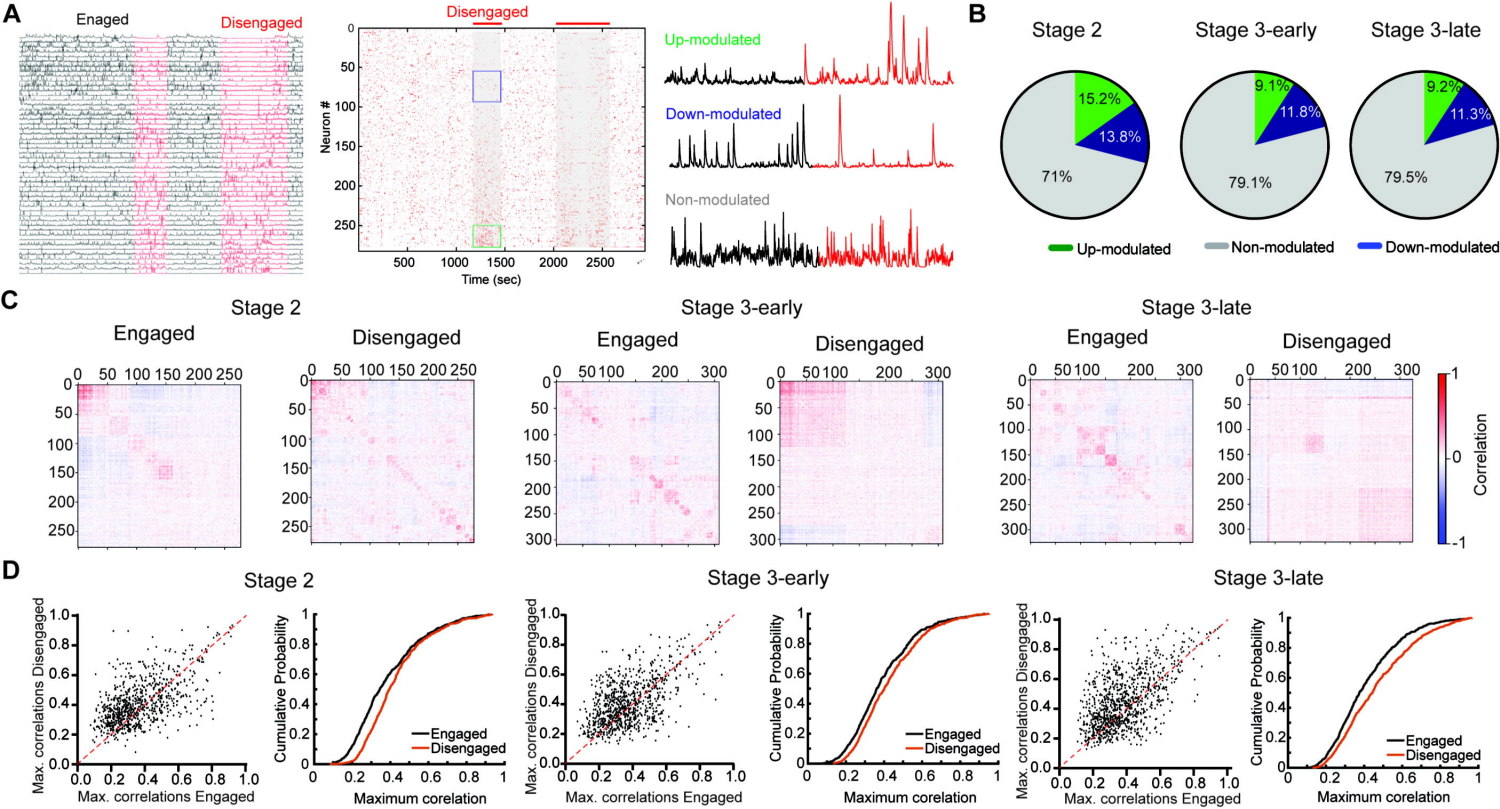
Calcium activity of PrL neurons during responding and non-responding periods. A) Calcium traces (left), raster plot (middle) showing calcium events across a recording session (black and red lines in the top show responding and non-responding periods, respectively) and calcium activity traces (right) from representative neurons which where up-modulated (top), down modulated (middle) and non-modulated (bottom) during non-responsive periods. B) Proportion of up-modulated (green), down-modulated (blue), and non-modulated (gey) neurons during recording sessions. C) Correlation matrices of neuronal activity from a representative mouse during rCPT recording sessions. D) Plots showing the average distribution of maximum correlation neuronal activity across engaged and disengaged states during rCPT recording sessions across all mice. Stage 2 engaged vs disengaged paired t test t(897)= 9.067 p≤ 0.001; Stage 3-early engaged vs disengaged paired t test t(961)= 7.408 p≤ 0.001; Stage 3-early engaged vs disengaged paired t test t(1109)= 12.6 p≤ 0.001.

To further characterize non-responsive periods of disengagement, we investigated whether calcium activity in PrL neurons reflects behavioral states. We first analyzed the averaged calcium activity of PrL neurons during non-responsive periods, which we compared to periods where mice were actively responding (engaged periods). We found heterogeneous responses in which calcium activity increased in some PrL neurons during non-responsive periods, while calcium activity decreased in others (Fig. 4A and Supplementary Fig. 5). The proportion of neurons whose activity was modulated during non-responsive periods was lower in Stage 3 sessions compared to Stage 2 (Stage 2 up-modulated= 136/898, down-modulated= 114/898; Stage 3-early up-modulated= 87/1020, down-modulated= 136/1020; Stage 3-late up-modulated= 102/1195, down-modulated= 125/1195) (Fig. 4B). Moreover, the overall calcium activity of neurons during non-responsive periods also decreased during Stage 3 sessions (Supplementary Fig. 5). Although the presence of up-modulated and down-modulated PrL subpopulations during non-responsive periods suggest the activation of different ensembles during engaged and disengaged states, it is possible that other changes at the network level that do not affect overall activity during behavioral states are occurring. To explore this idea, we assessed the network state of the PrL by analyzing the correlation between the calcium activity of PrL neurons during responsive and non-responsive periods. We found different correlation profiles across different mice during responsive and non-responsive periods (Supplementary Fig. 6), but with the overall effect of a lower correlation index in the PrL calcium activity during non-responsive periods (Stage 2 engaged ^mean max corr index^= 0.385 ± 0.006, disengaged ^mean max corr index^ = 0.428 ± 0.006; Stage 3-early engaged ^mean max corr index^= 0.393 ± 0.005, disengaged ^mean max corr index^ = 0.427 ± 0.005; Stage 3-late engaged ^mean max corr index^= 0.4080 ± 0.005, disengaged ^mean max corr index^ = 0.466 ± 0.006) (Fig. 4D). All together, these results lend evidence to the hypothesis that PrL calcium activity and PrL network state reflect changes in behavioral states during rCPT behavior.

## Discussion

In this study, we used *in vivo* calcium imaging to characterize activity patterns of PrL neurons during sustained attention in the rCPT. Our results revealed heterogeneous responses of PrL neurons across behavior, with a higher proportion of PrL neurons modulated during correct responses, especially in proficient sessions (Stage 2-early and Stage 3-late). These results are in line with previous electrophysiological studies using single unit recordings in fully trained mice that showed a higher proportion of modulated PrL neurons during trials that ended in correct responses [29,31,36]. Like other attention tasks, the rCPT assesses attention by determining the animal’s ability to discriminate between stimuli over extended sessions where at least one stimulus is paired with reward [13]. In addition to sustained attention [17,29,30,33,38], the PrL is linked to stimulus discrimination [39,40,41] and reward processing [42,43,44], which are important for rCPT performance. The peak of PrL calcium transients occurred between the stimulus presentation and directly following screen-touch, which could reflect PrL computations for visual discrimination, decision making, and reward expectation, all of which are consistent with a role for the PrL in attention-related sensory processing [39,45,46]. Unlike previous studies that assessed neuronal patterns in attention tasks in fully trained animals [29,30,31,33,38,47], we analyzed PrL calcium activity at different points of rCPT training that required varying levels of cognitive demand and proficiency. PrL calcium activity differentiates between response types across recording sessions, which is in line with our data demonstrating increased PrL delta and theta power during correct responses across different stages of rCPT training [34]. Here, the proportion of modulated PrL neurons during correct responses increased as rCPT training progressed. Together, the data suggests that calcium activity in PrL neurons and LFPs in the PrL track stimulus discrimination, decision making, and response outcome across learning states during periods of certainty and uncertainty [48,49,50].

Although the proportion of PrL neurons that were modulated during incorrect responses was small, we did identify a subset whose calcium activity peaked close to the time of stimulus presentation. Previous reports linked activity in the human ACC with conflict monitoring and response inhibition [51,52,53,54]. Specifically, event-related potentials show increased ACC activity prior to non-target trials [52,53,55] even in trials that end in incorrect responses [53]. In rodents, lesioning [15,56] or inactivating the PrL [57] impairs accuracy, and results in a disinhibited response profile on attention tasks. Moreover, inhibiting the PrL decreases the ability of rats to resolve conflict between competing responses [58]. Together, these reports point to the PrL as a point of convergence for conflict monitoring and response inhibition. [56,57]. Those PrL neurons that increase their activity prior to incorrect responses may reflect competition between execution and response inhibition, a mechanism that may be processed in downstream regions via axonal projections of PrL projection neurons. Strengthening this hypothesis, several PrL target regions, including the nucleus accumbens [59], thalamus [60], and LC [33,61,62], are implicated in conflict monitoring and response inhibition. In addition, we recently reported that changes in LFPs recorded in the PrL predict changes in LC LFPs within gamma frequencies during incorrect responses [63]. Given the possible role of PrL-LC projectors in cognitive control and response inhibition, future experiments aim to assess changes in the activity patterns of LC-projecting PrL neurons during incorrect responses across behavioral sessions in the rCPT. These experiments would better resolve the role of PrL-LC circuit dynamics in encoding cognitive control and response inhibition during sustained attention.

### PrL activity patterns during attention lapses

Although peri-event analysis during correct and incorrect responses has provided important information about the role of specific brain regions and their activity patterns during processes related to sustained attention [29,34,63], moment-to-moment analysis of neuronal activity patterns during attentional lapses would be invaluable to better understand cellular mechanisms. To further investigate PrL calcium activity during attention lapses, we identified long, non-responsive periods that represent task disengagement during rCPT behavior. To our knowledge, this is the first time attentional lapses and neuronal activity patterns associated with these lapses were assessed in the rCPT. Our results showed that individual mice intercalate engaged and disengaged periods across rCPT sessions with characteristic patterns. Moreover, in sessions requiring higher cognitive demand (Stage 3), the number of disengaged periods increased while the number of rewards obtained before the first disengaged period decreased. This is in line with the “overload theory”, which explains attentional lapses as a consequence of resource depletion and cognitive fatigue [64,65,66,67]. Previous reports using other translational attention tasks have linked attentional lapses to omissions [68,69,70,71,72], and PrL activity patterns during omissions are similar to those seen during incorrect responses [29,31]. Attention lapses may involve changes in neuronal states [73] Therefore averaging omission trials to investigate neuronal activity underlying attention lapses could limit the ability to assess changes in neuronal states, as it overlooks moment-to-moment fluctuations in neuronal activity, especially if those fluctuations occurred during ITIs [74]. Here, we identified disengaged periods, which contain a relatively high number of continuous trials that end in omissions, to determine moment-to-moment changes in neuronal states related to attention lapses. We observed single-cell level modulations of PrL neuronal activity during engaged and disengaged periods, which could reflect activation of ensembles during sustained attention and attention lapses. Moreover, we showed population-level changes in the degree of correlated activity of PrL neurons during engaged and disengaged periods. These findings are in line with other data showing that attention causes cortical desynchronization by decreasing inter-neuronal correlation [75,76]. Assessing the role of different neurotransmitter systems in driving the changes in the correlated activity during attention and attention lapses could help to determine the cellular and molecular mechanisms underlying sustained attention. Norepinephrine (NE) signaling within the PrL-LC circuit is particularly interesting in this context. The PrL sends projections to the LC, which modulate noradrenergic transmission [77,78]. The PrL also receives noradrenergic inputs from the LC, which have a modulatory effect on attention [79,80,81]. NE released in the cortex facilitates synchronization during attention-related behaviors [82,83], and denervating noradrenergic inputs to the PrL decreases arousal and wakefulness in novel environments where attentional resources should be allocated [84]. Moreover, changes in LC-NE neuron activity are associated with task disengagement [85] as well as attentional lapses [83]. Better understanding regulation of bidirectional activity in the PrL-LC circuit, and the effect of NE release on neuronal activity in PrL neurons during attentional lapses could advance our understanding on mechanisms underlying sustained attention.

## Supplementary Material

Supplement 1
