# Supplementary figures and images for: Patterns of neural activity in prelimbic cortex neurons correlate with attentional behavior in the rodent continuous performance test
Source: bioRxiv. 2025 Sep 11:2024.07.26.605300. Originally published 2024 Jul 26. Preprint. [Version 2] doi: 10.1101/2024.07.26.605300 (PMC11291163; doi:10.1101/2024.07.26.605300)

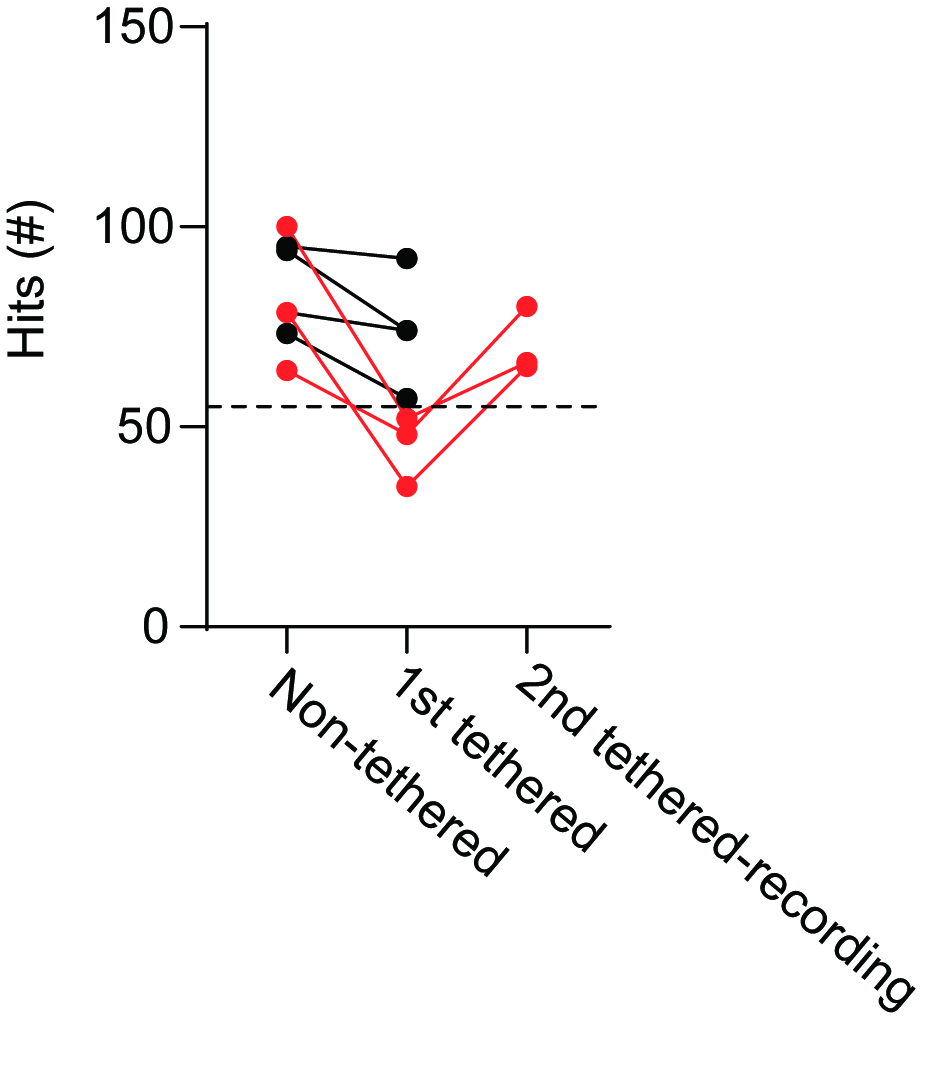

Supplement: Supplement 1 — Supplementary Fig. 1. Effect of tethering on behavior during Stage 2 training sessions. Number of responses during a non-tethered session and tethered sessions. Mice represented by orange circles decreased their responses under criteria threshold (55 responses) during the first tethered session, and required an extra tethered session to reach criteria for recording. [file media-1.tif]

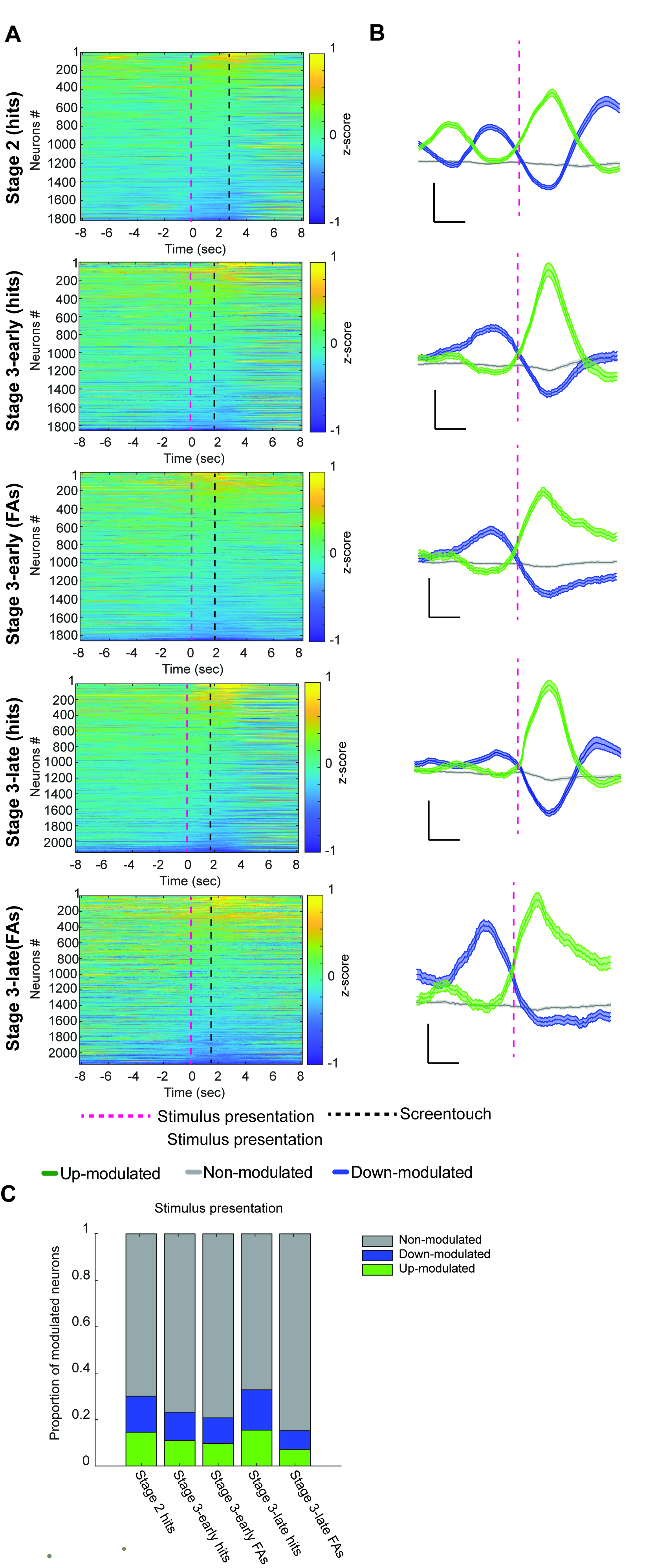

Supplement: Supplement 2 — Supplementary Fig. 2. Peri-event analysis of PrL calcium activity during rCPT aligning events to stimulus presentation . A) Heat maps showing normalized change in calcium activity (z-scored) surrounding hits (correct responses) and false alarms (FAs, incorrect responses) during rCPT recording sessions after aligning events to stimulus presentation. B) Calcium activity traces from up-modulated (green), down-modulated (blue) or non-modulated (gray) neurons surrounding the behavioral response. The pink dotted line represents the stimulus presentation Scale bar: 2 s, and z-score= 0.2. C) Bar graphs showing proportion of neurons that were up-modulated (green), down-modulated (blue) or non-modulated (gray) surrounding the behavioral response. Stage 2 hits up-modulated = 14.5% (265/1822), down-modulated = 15.53 (283/1822), non-modulated = 69.92% (1274/1822); Stage 3-early hits up-modulated = 11.0% (204/1860), down-modulated = 12.2% (227/1860), non-modulated = 76.83% (1429/1860; Stage 3-early FAs upmodulated = 9.7% (180/1860), down-modulated = 11.1% (206/1860), and non-modulated = 79.3% (1474/1860); Stage 3-late hits upmodulated = 15.4% (331/2146), down-modulated = 17.4% (373/2146), non-modulated = 67.2% (1442/2146); Stage 3-late FAs up-modulated = 7.2% (154/2146), down-modulated = 8.1% (173/2146), non-modulated = 84.8% (1819/2146). [file media-2.tif]

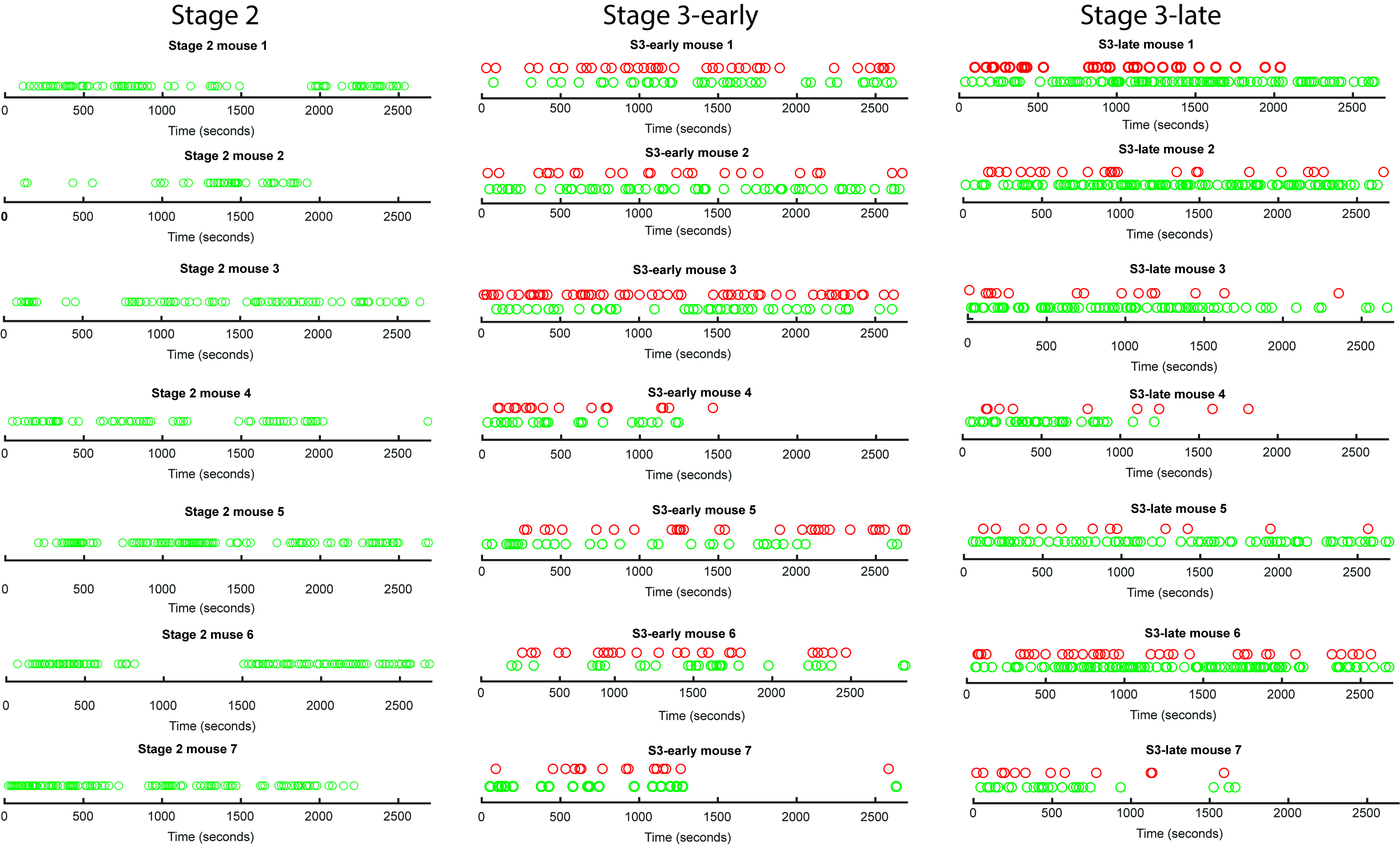

Supplement: Supplement 3 — Supplementary Fig. 3. Responses across time during rCPT recording sessions. Distribution of responses (hits- green circles and FAs- red circles) during 45 min (2700 s) recordings sessions. [file media-3.tif]

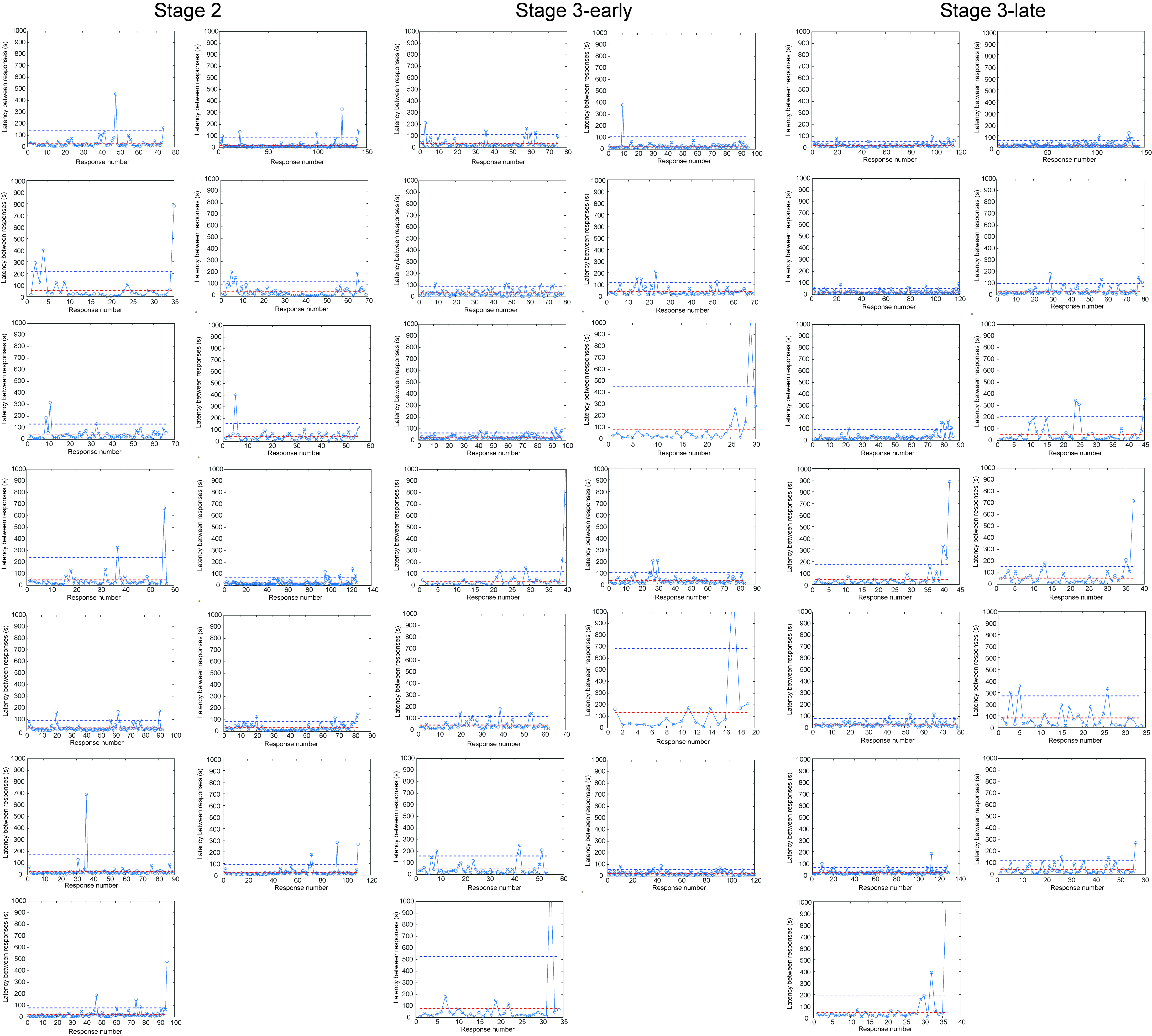

Supplement: Supplement 4 — Supplementary Fig. 4. Latency between responses during rCPT recording sessions. Plots showing latency between responses during rCPT recording sessions. Red dotted line shows average latency between two responses for that particular mouse and session. Blue dotted line shows the threshold for disengagement periods (mean + 2DTD) for that particular mouse and session. [file media-4.tif]

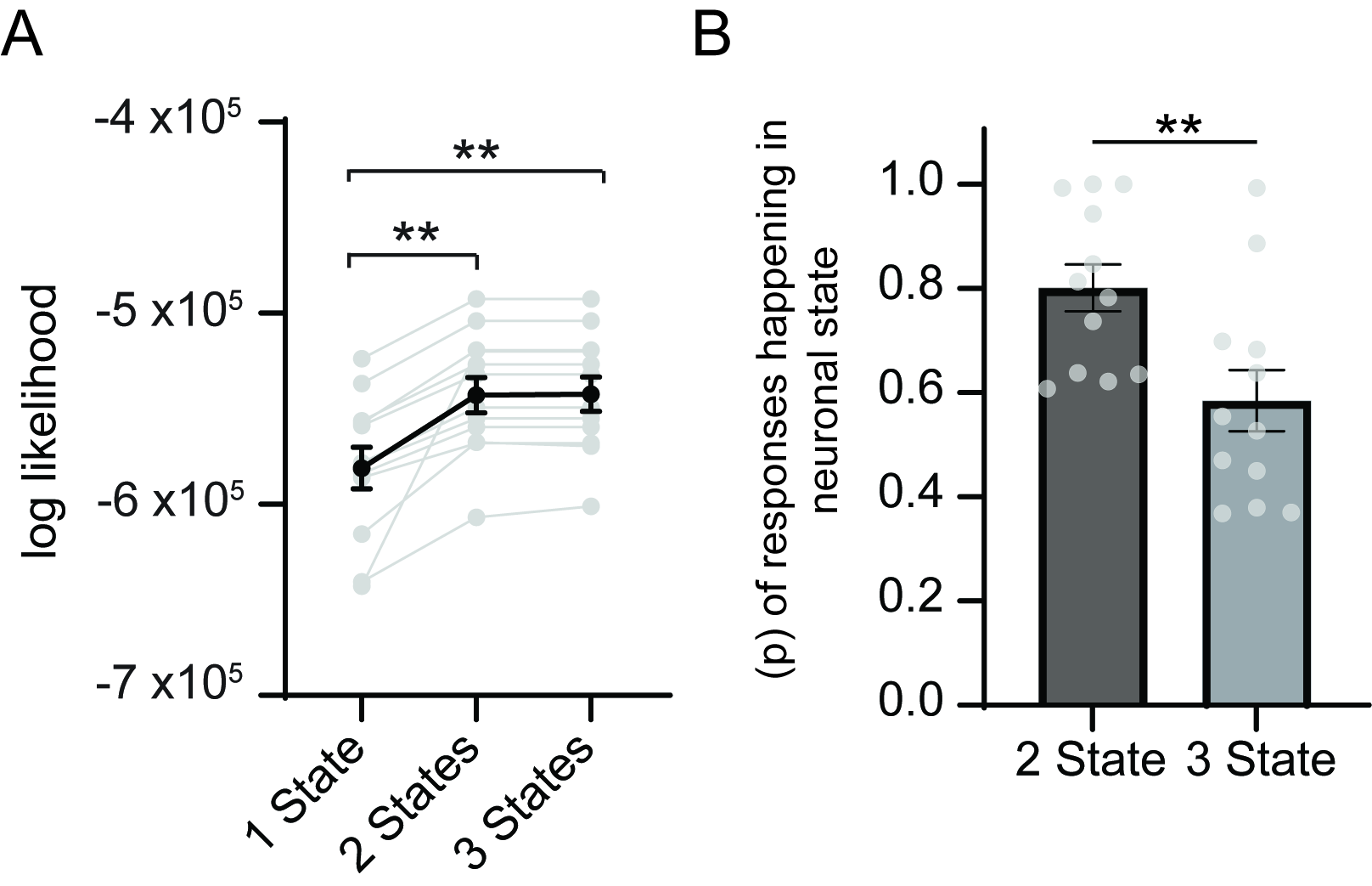

Supplement: Supplement 5 — Supplementary Fig. 5. Two-state HMM performance. A) Graph showing the log-likelihood of HMMs fitted with one, two, or three states to neural activity data. Models with two and three states showed significantly higher log-likelihoods compared to the one-state model. No statistical difference was observed between the two- and three-state models. Repeated measures ANOVA F= 23.16 p ˂ 0.001 Tuckey’s posthoc test 1 state vs 2 states p= 0.001, 1 state vs 3 states p= 0.001, 2 states vs 3 states p= 0.822. B) Bar graph showing the probability of responses occurring in the dominant state for the two- and three-state HMMs. The two-state HMM yielded a higher probability of responses in the dominant state compared to the three-state model (t22= 2.94 p= 0.008 [file media-5.tif]

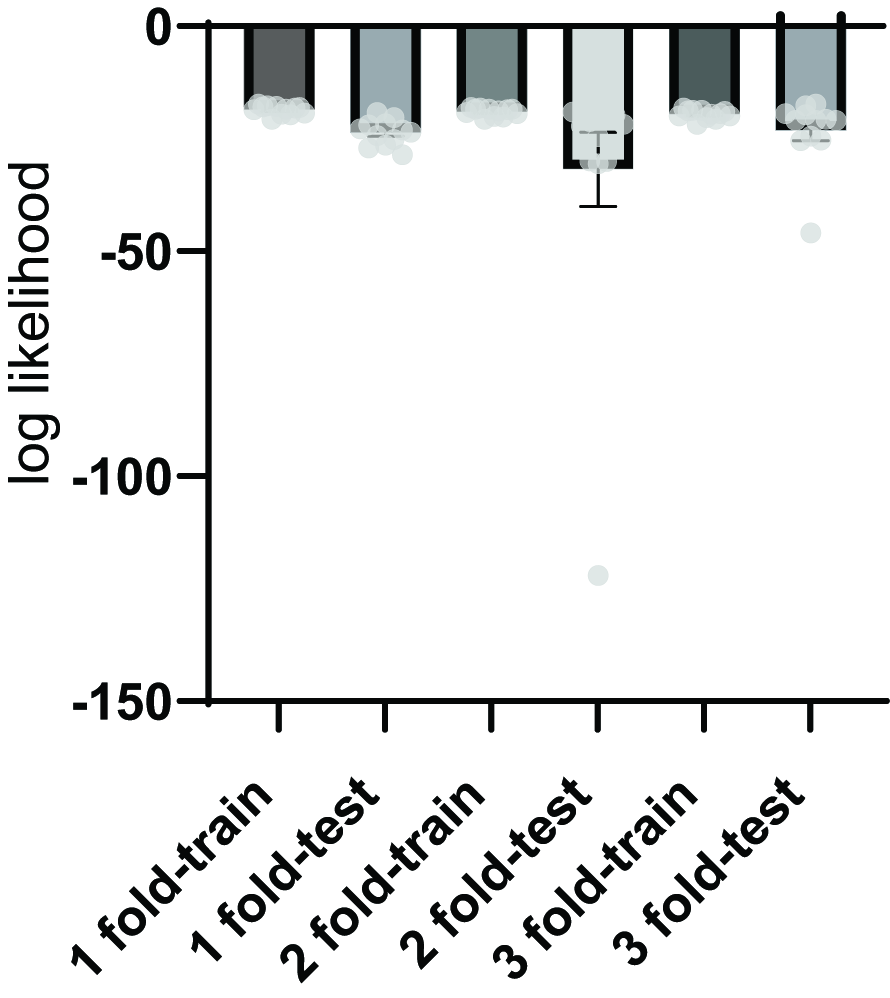

Supplement: Supplement 6 — Supplementary Fig. 6. Two-state HMM log-likelihood computed using 3-fold cross-validation within individual behavioral sessions. Sessions were divided into four equal segments, and train versus test sets were defined corresponding to fold number (1-fold; training = first 25% of data, test = second 25% of data). No significant differences in log likelihood across different folds between train and test session. ANOVA F5,66 = 1.492 p = 0.2043. [file media-6.tif]

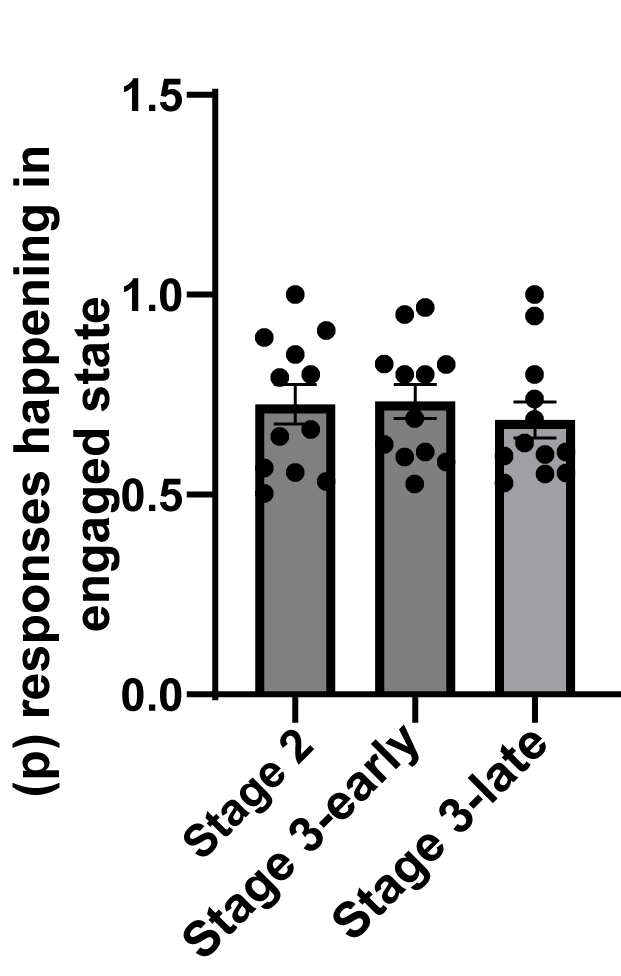

Supplement: Supplement 7 — Supplementary Fig. 7. Probability of engaged state across rCPT stages. No statistical difference in the probability of the dominant state given responses between S3-early and S3-late (t22= 0.7413 p=0.4663) [file media-7.tif]

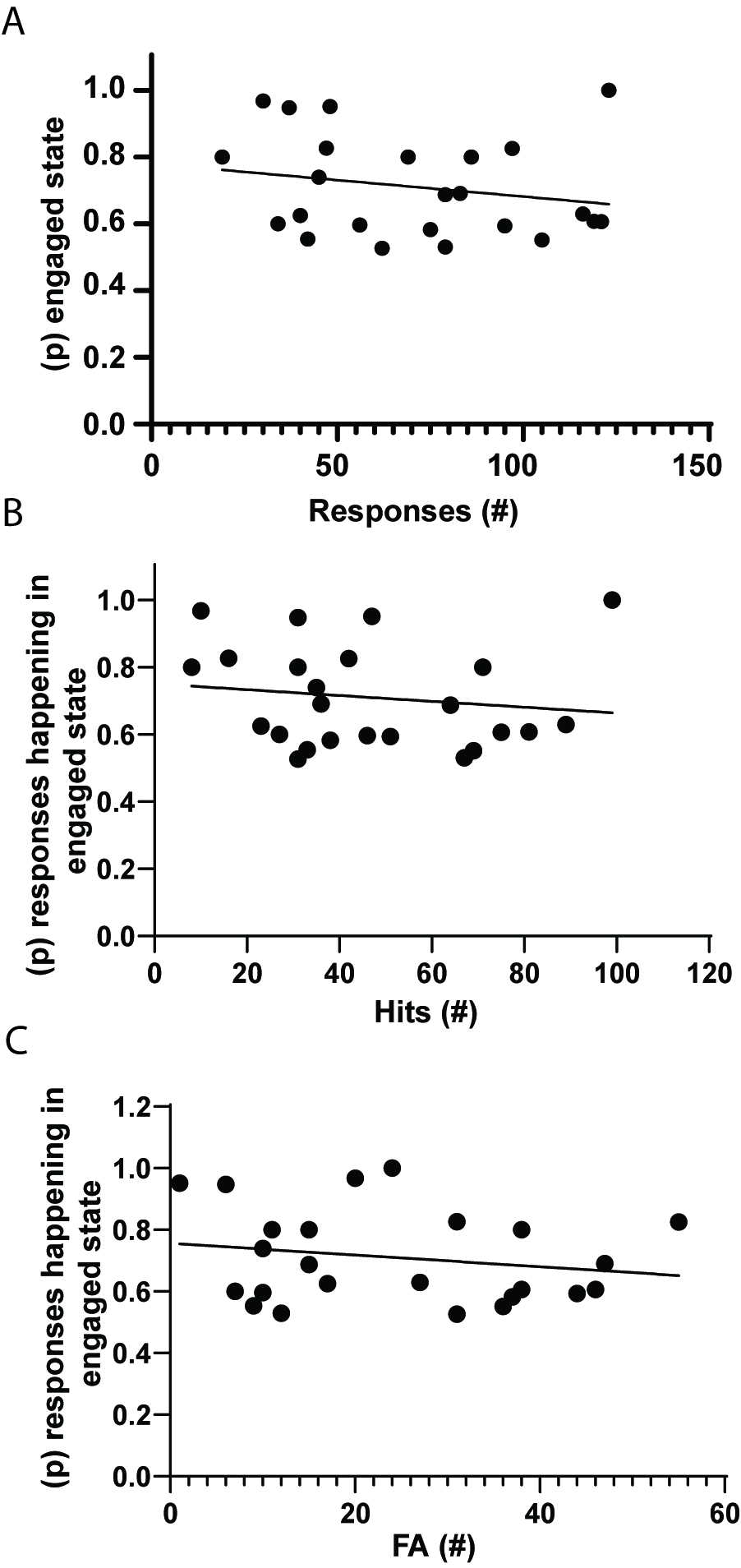

Supplement: Supplement 8 — Supplementary Fig. 8. Correlation analysis between the probability for the engaged state against hits and FAs number. A) Correlation graphs showing Bayesian probability of the engaged state given total number of responses. There is no statistical correlation between the number of responses and the probability of responses happening in the engaged state (pearson r= −0.207, p= 0.331). B) Correlation graphs showing Bayesian probability of the engaged state given the number of hits. There is no statistical correlation between the number of hits and the probability of responses happening in the engaged state (pearson r= −0.144, p= 0.502). Correlation graphs showing Bayesian probability of responses happening in the engaged state over the number of FAs. There is no statistical correlation between the number of FAs and the probability of FAs happening in the engaged state (pearson r= −0.194, p= 0.363). [file media-8.tif]

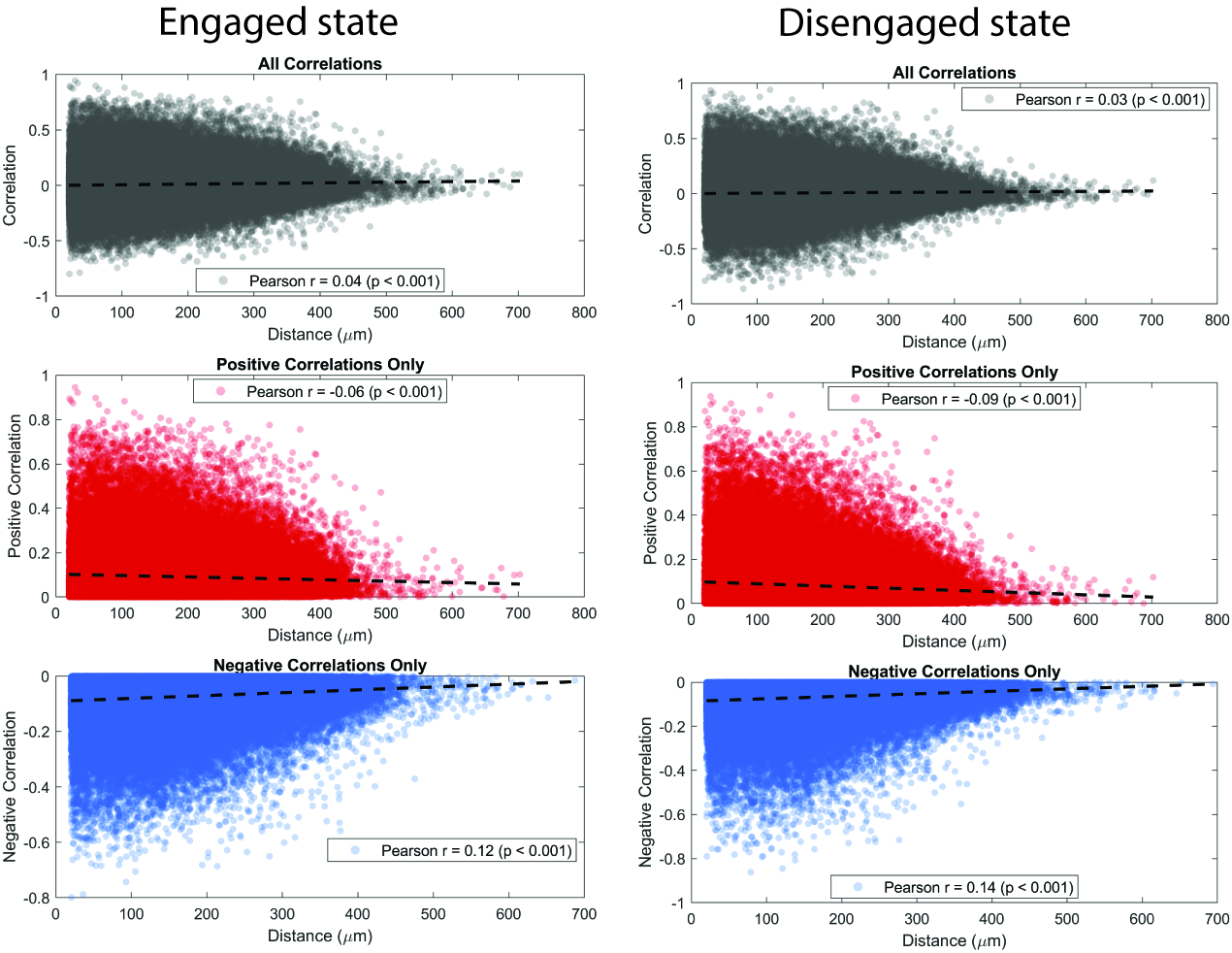

Supplement: Supplement 9 — Supplementary Fig. 9. Correlated activity between pairs of PrL neurons decreases with distance across neuronal states associated with task engagement. Analysis of neuronal activity correlation between pairs of neurons and distance during dominant (right) and non-dominant state. [file media-9.tif]

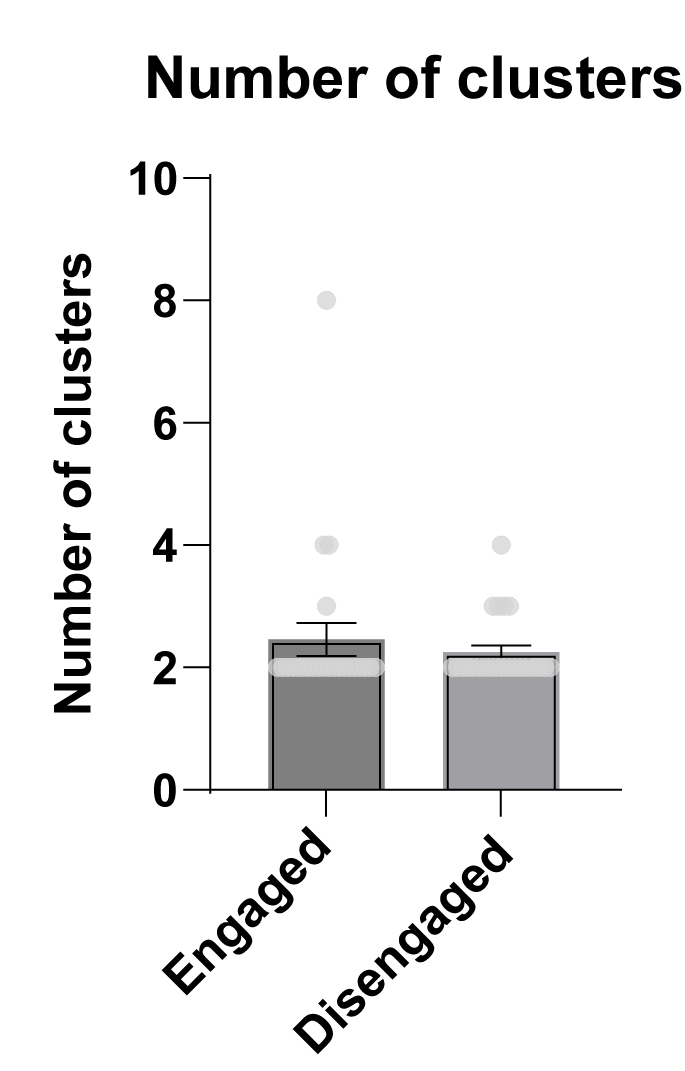

Supplement: Supplement 10 — Supplementary Fig. 10. k=2 is the best parameter for k-means cluster analysis during neuronal states across sessions. Bar graph showing the results of Silhouette analysis on correlated activity during rCPT sessions for posterior K-means cluster analysis. There is no significant differences in the number of clusters across engaged and disengaged state (t23= 0.7068 p= 0.4868). [file media-10.tif]

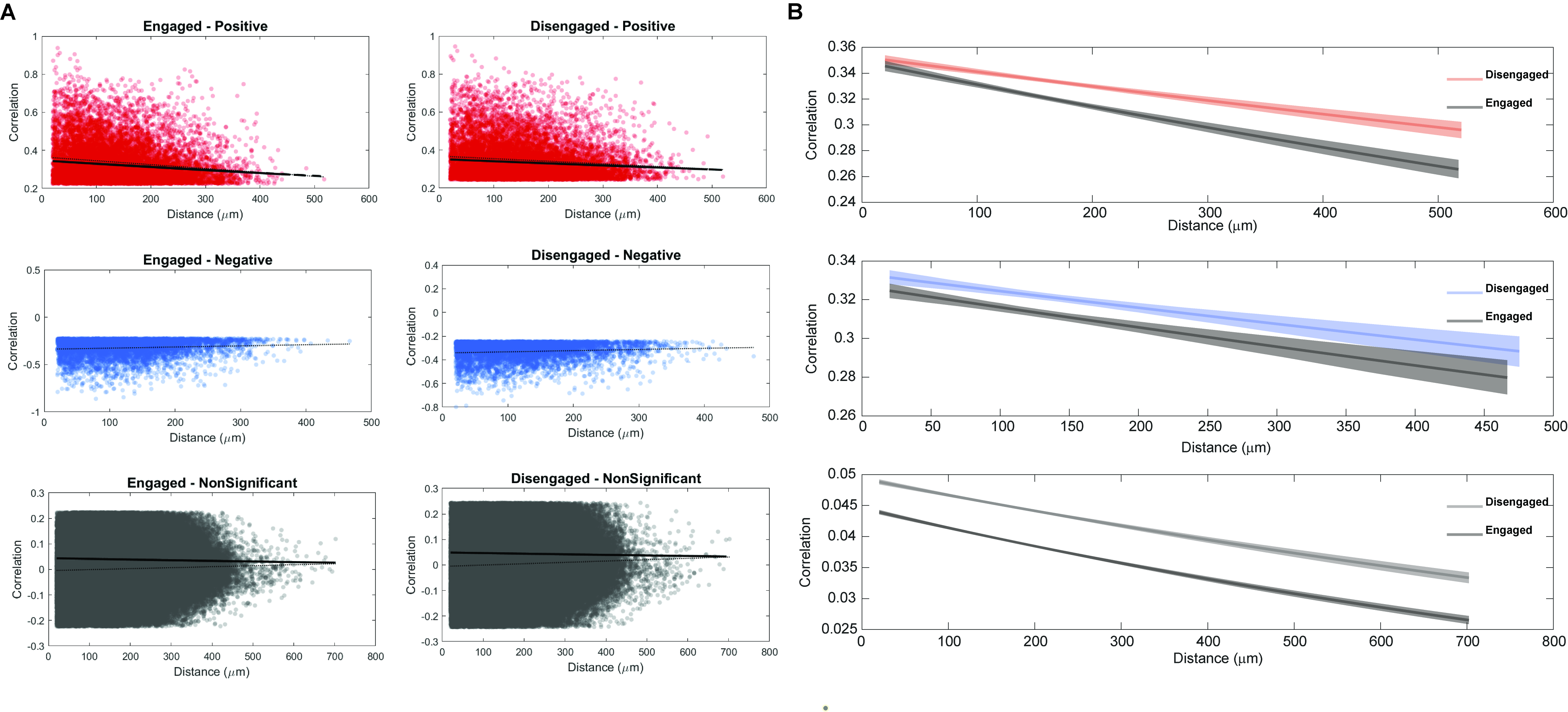

Supplement: Supplement 11 — Supplementary Fig. 11. Correlated activity between pairs of PrL neurons decreases with distance across neuronal states associated with task engagement. A) Analysis of neuronal activity correlation between pairs of neurons and distance during (Engaged (right) and Disengaged state divided into significant positive correlated, significant negative-correlated, and non-significant correlated neurons. B) Graphs showing fit of an exponential decay model used to examine correlation strength decline with distance. Decay of positive correlations was significantly steeper in the Engaged state compared to the Disengaged state (z = 4.21, p˂ 0.001). Non-significant correlations also showed a significantly steeper decay in the Engaged state (z = 5.24, p˂ 0.001). Negative correlations did not differ significantly between states (z = 1.13, p = 0.257). [file media-11.tif]
