# Supplementary material for: Patterns of neural activity in prelimbic cortex neurons correlate with attentional behavior in the rodent continuous performance test
Source: bioRxiv. 2024 Jul 26:2024.07.26.605300. Preprint. [Version 1] doi: 10.1101/2024.07.26.605300 (PMC11291163; doi:10.1101/2024.07.26.605300)
Supplement: Supplementary file 1 [file NIHPP2024.07.26.605300v1-supplement-1.pdf]

bioRxiv preprint doi: https://doi.org/10.1101/2024.07.26.605300; this version posted July 26, 2024. The copyright holder for this preprint (which was not certified by peer review) is the author/funder, who has granted bioRxiv a license to display the preprint in perpetuity. It is made available under aCC-BY 4.0 International license.

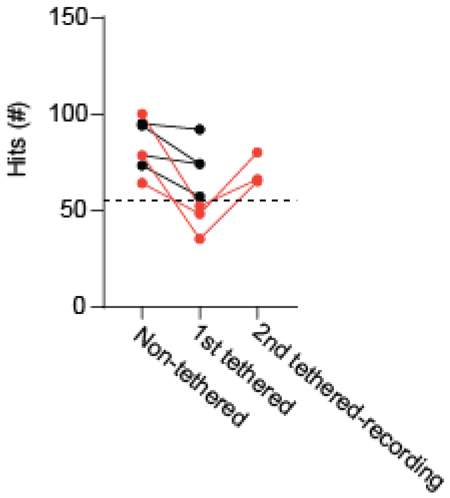

Supplementary Fig. 1. Effect of tethering on behavior during Stage 2 training sessions. Number of responses during the previous non-tethered session and during the tethered sessions. Mice represented by orange circles decreased their responses under criteria threshold (55 responses) during the first tethered session, and required an extra tethered session to reach criteria for recording. .

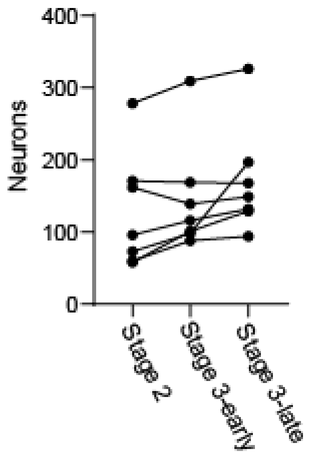

Supplementary Fig. 2. Number of neurons detected during recording sessions. Number detected PrL neurons across stage 2, stage 3-early, and stage 3-late recording sessions. A higher number of PrL neurons was detected in 5/7 mice.

bioRxiv preprint doi: https://doi.org/10.1101/2024.07.26.605300; this version posted July 26, 2024. The copyright holder for this preprint (which was not certified by peer review) is the author/funder, who has granted bioRxiv a license to display the preprint in perpetuity. It is made available under aCC-BY 4.0 International license.

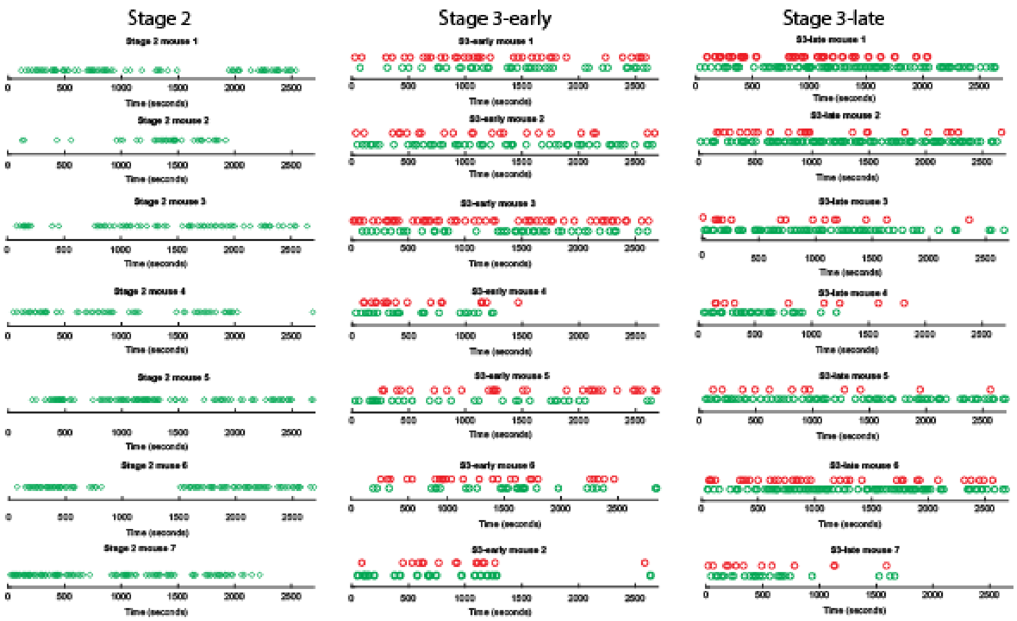

Supplementary Fig. 3. Responses across time during rCPT recording sessions. Distribution of responses (hits- green circles and FAs- red circles) during 45 min (2700 s) recordings sessions.

bioRxiv preprint doi: https://doi.org/10.1101/2024.07.26.605300; this version posted July 26, 2024. The copyright holder for this preprint (which was not certified by peer review) is the author/funder, who has granted bioRxiv a license to display the preprint in perpetuity. It is made available under aCC-BY 4.0 International license.

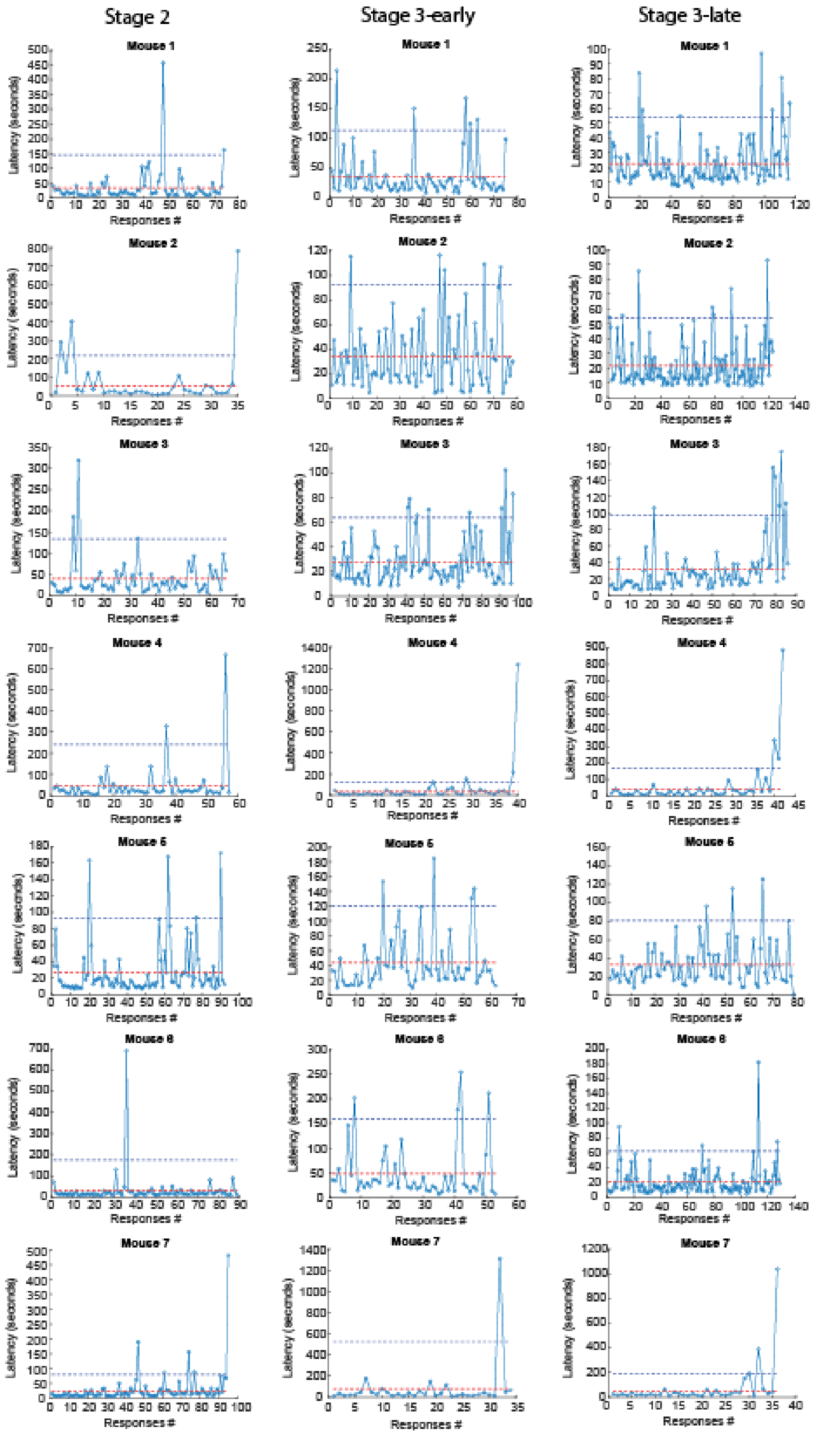

Supplementary Fig. 4. Latency between responses during rCPT recording sessions. Plots showing latency between responses during rCPT recording sessions. Red dotted line shows average latency between two responses for that particular mouse and session. Blue dotted line shows the threshold for disengagement periods (mean + 2DTD) for that particular mouse and session.

bioRxiv preprint doi: https://doi.org/10.1101/2024.07.26.605300; this version posted July 26, 2024. The copyright holder for this preprint (which was not certified by peer review) is the author/funder, who has granted bioRxiv a license to display the preprint in perpetuity. It is made available under aCC-BY 4.0 International license.

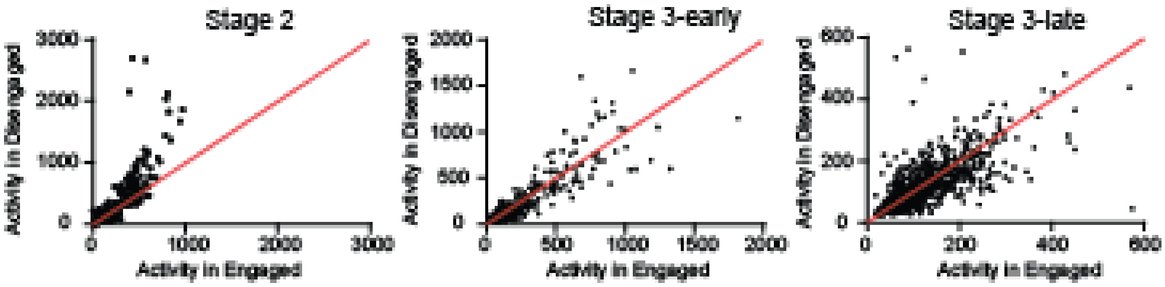

Supplementary Fig. 5. Scatter plots showing the average activity of each recorded neuron during responding and non-responding periods

bioRxiv preprint doi: https://doi.org/10.1101/2024.07.26.605300; this version posted July 26, 2024. The copyright holder for this preprint (which was not certified by peer review) is the author/funder, who has granted bioRxiv a license to display the preprint in perpetuity. It is made available under aCC-BY 4.0 International license.

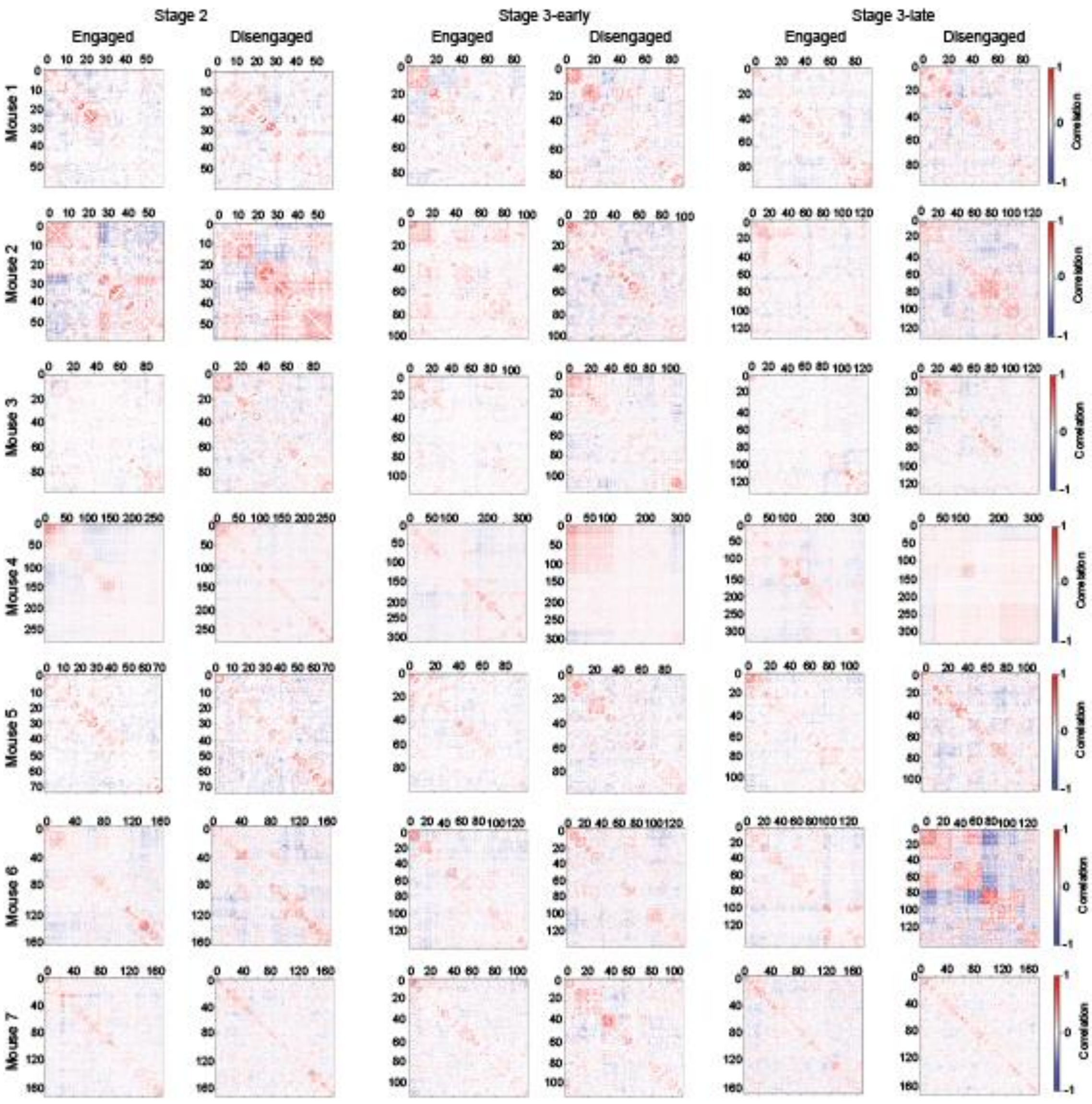

Supplementary Fig. 6. Calcium activity correlations during engaged and disengaged behavioral states. Correlation matrices of neuronal activity from a representative mouse during rCPT recording sessions during Stage 2, Stage 3-early, and Stage 3-late .
